# Synergistic effects of light and plasma catalysis on Au-modified TiO_2_ nanotube arrays for enhanced non-oxidative coupling of methane†

**DOI:** 10.1039/d5cy00206k

**Published:** 2025-05-01

**Authors:** Victor Longo, Luana De Pasquale, Siglinda Perathoner, Gabriele Centi, Chiara Genovese

**Affiliations:** a Department of Chemical, Biological, Pharmaceutical and Environmental Sciences, ERIC aisbl and CASPE/INSTM, University of Messina Viale F. Stagno D'Alcontres 31 98166 Messina Italy chiara.genovese@unime.it

## Abstract

The direct conversion of methane into value-added hydrocarbons represents one of the most energetically efficient pathways to shift industrial processes towards more sustainable low-carbon technologies. In this study, we present a novel planar dielectric barrier discharge (DBD) reactor equipped with a quartz window for catalyst illumination, to explore potential synergies between light and plasma in the catalytic non-oxidative coupling of methane (NOCM) reaction. Highly ordered TiO_2_ nanotube arrays were grown on a Ti mesh and further modified with Au nanoparticles to improve light absorption and reactivity, thereby acting as the DBD electrode and NOCM catalyst. The introduction of gold significantly enhances performances, achieving a substantial rise of 64% in plasma-assisted methane conversion compared to the bare support while shifting the selectivity towards alkanes and C_3+_ hydrocarbons. For the first time, we demonstrated the effect of light irradiation and its interaction with plasma, revealing an effective synergistic mechanism between plasma and light in gold-modified materials. Notably, a 21.5% increase in the intrinsic rate of the NOCM surface process under irradiation was achieved. This improvement is attributed to two factors: induced physical changes in the nature of the plasma micro-discharges and the creation of specific surface vibrational states on the catalyst.

## Abstract 2

This study presents an effective synergistic mechanism between plasma and light in gold-modified TiO_2_ materials for the direct conversion of methane into value-added C_2+_ hydrocarbons.

## Graphical abstract

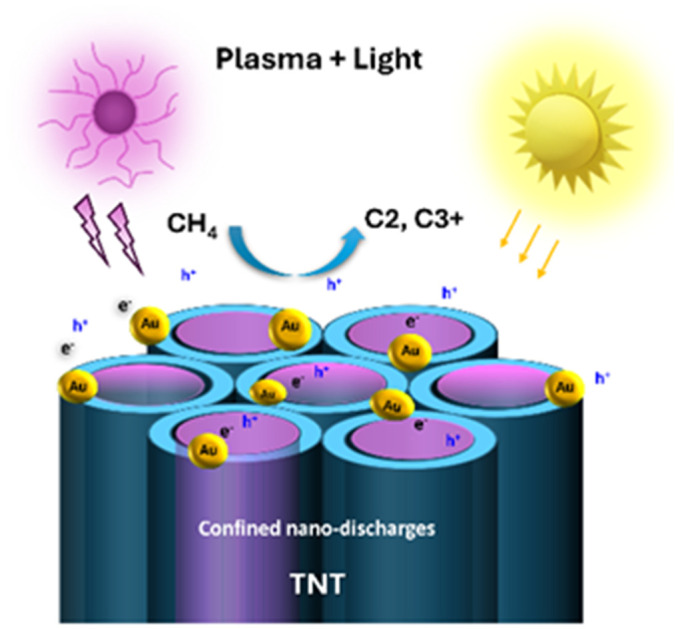

## Introduction

Recently, significant efforts have been directed toward addressing the rising demand for clean energy by converting and upgrading small molecules. Methane (CH_4_), being an abundant and clean fossil fuel with high specific energy compared to other hydrocarbons, represents a great opportunity for obtaining fuels or high-value chemicals, particularly when the conversion is a photo-driven process.^1,2^ However, the controlled activation of the C–H bond(s) in methane remains a crucial challenge.^3,4^

The industrial conversion of methane typically follows an indirect route that requires oxidants to facilitate C–H bond activation. This process involves reforming methane into syngas (CO + H_2_) through water or carbon dioxide reactions at elevated temperatures. Syngas is subsequently utilised to produce C_2_–C_4_ hydrocarbons (*via* the Fischer–Tropsch process), methanol and other products. Methanol can then be catalytically converted to olefins, aromatics or other chemicals.

The controlled oxidation of methane to methanol using Cu-zeolites or H_2_O_2_ as oxidant^5–7^ presents significant challenges; however, reports indicate that the productivity remains extremely low. The intensively studied reaction of oxidative conversion to C_2+_ hydrocarbons (OCM)^8^ gives better yields. However, the reaction is not exploitable due to the high temperatures required and the difficulty in scaling up. Recently, studies addressing direct methane conversion have been focused on the use of technologies based on renewable energy, *e.g.* photo-^9,10^ and electro-catalysis^11,12^ or plasma catalysis.^13–16^

With the rise of environmental problems, there is in fact, an increasing need to shift industrial processes toward more sustainable low-carbon technologies. Many studies have explored alternative methods to overcome the issue of the strong bond energy of C–H in methane (434 kJ mol^−1^), which requires high temperatures to be activated.^17^ Among these alternatives, the direct non-oxidative conversion of methane to hydrocarbons has been widely recognised as the potentially most energetically efficient path, even if still challenging.^4,11,18,19^

A promising technology in this context is plasma-assisted methane conversion. In the plasma zone, accelerated high-energy electrons may activate the methane molecules at mild temperatures by generating highly reactive species (radicals, ions, *etc.*).^20,21^ Methane conversion efficiency in plasma-assisted reactions depends on various experimental conditions, such as plasma power, reactor design, and feed gas composition. However, selectivity remains insufficient, often resulting in the significant formation of unwanted carbonaceous by-products.^20,22–24^ Combining plasma technology with catalysis may modulate the selectivity and reduce carbon deposits.^25^

An alternative challenging approach is the light-assisted coupling of methane.^1,26–28^ In this case, the C–H bond activation is performed by the charge carriers generated by light (the cleanest energy source). Despite its potential, the yields in CH_4_ coupling remain insufficient for industrial/commercial upscaling.^19,29^ On the other hand, plasma technology is a very effective and straightforward approach for converting “hard-to-activate” molecules at mild temperatures, representing an efficient solution for electrifying chemical processes. While synergies between photocatalytic and electrocatalytic processes have been recognised for some time,^30,31^ the potential synergic effects between photocatalysis and plasma catalysis have yet to be thoroughly explored.^32^

From a theoretical perspective, such synergies are plausible. Still, only a few examples exist in the literature, *i.e.* for VOC conversion, indicating high efficiency through UV light combined with TiO_2_ in a dielectric barrier discharge (DBD) tubular reactor.^33,34^ One example is reported on the use of plasma and light processes for CO_2_ reduction, but conducted in two consecutive steps.^35^ However, to the best of our knowledge, no studies have documented synergistic effects between light and plasma catalysis specifically for methane non-oxidative upgrading, which is the primary objective of this study.

We recently demonstrated that the rate of ethane production in photocatalytic processes is strongly influenced by charge-transfer processes and correlated to the concentration of methyl radicals.^28^ Thus, in theory, it is expected to significantly enhance the concentration of radical species even in a low-energy plasma. In addition, light can modify the surface states of the (photo)catalyst to facilitate the activation of vibrationally excited molecules. Because of light absorption, charged species reinforce the surface polarisation and generate locally enhanced electric fields to enrich the number of reactive species within the plasma environment. Xu *et al.*^36^ recently showed that a photon–phonon coupling, *e.g.* between lattice vibrations and charges generated by the light absorption, significantly enhanced the methane aerobic conversion to formaldehyde at 150 °C.

Finally, light modifies the dielectric properties of a photocatalyst by increasing the dielectric constant, which may also be seen as a potential extra benefit for plasma, *e.g.* inducing changes in the type of discharges (*e.g.*, inside the catalyst pores^37^ or from filament-type to multiple surface-confined micro-discharges^38,39^). The light originating from plasma radiative de-excitation is too sparse to irradiate effectively the photocatalyst, as reported in some studies that analysed the use of a photocatalyst in combination with NTP.^40,41^ Thus, an external source is necessary, along with a new design for the plasma reactor. Here, we proposed and realised a novel planar DBD reactor equipped with a quartz window for illumination. The main objective is to investigate the potential synergies between light and plasma, proving the real feasibility of coupling photo- and plasma-catalysis in the non-oxidative coupling of methane (NOCM).

An electrode that also acts as a catalyst has been chosen for these investigations, consisting of a metallic Ti mesh that has been anodised to create a layer of well-organised TiO_2_ nanotube (TNT) arrays, known for their remarkable photocatalytic activity and suitability for electro-photocatalytic processes.^42^

TiO_2_ nanotubes were selected for their significant synergistic effects in integrating photo- and plasma-catalytic processes.^43^ Under light irradiation, the organised geometry of the nanotubes offers an ideal vectorial path for the conduction of photogenerated electrons,^44^ while enhancing the surface polarity of the materials.^45,46^ Furthermore, the well-structured nanotube arrays provide several advantages for creating active species in the plasma. First, with their geometry, the nanotube edges (and walls) strongly enhance the electric field.^46,47^ Second, the plasma can be confined in the mesopores of the nanostructures,^37,48,49^ promoting the catalyst plasma interaction^20,22^ and micro-discharges.^21^ The TNT/Ti mesh was further modified with gold nanoparticles.

Au on titania has indeed been previously shown to drive the methane coupling reaction with light, under both aerobic^10,50^ and anaerobic conditions.^10,27^ Au nanoparticles on titania also induce plasmonic effects by light absorption, *e.g.* the generation of hot charges.^51–53^ Although other metals, like Pd or Pt, may offer a greater solution for C–H bond activation, previous studies indicate that among several metals (Au, Pd, Ag, and Cu) deposited on the TNT/Ti mesh, Au provides the greatest photo-response^44^ due to the surface plasmon resonance (SPR) effect. Therefore, it is likely to have the greatest photo-enhanced effect when associated with plasma.^54^

These advantages may be amplified under light irradiation through the creation of surface states that further enhance surface polarisation.^55^ The above concepts are summarised in Fig. S1,† that illustrates schematically how the use of Au-modified TiO_2_ nanotube arrays can improve CH_4_ upgrading in a plasma-photo process.

## Results and discussion

### Characterisation of the electrode

**Fig. 1:**
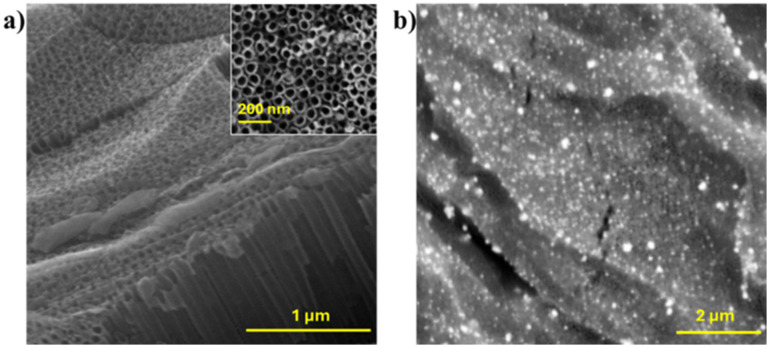
Scanning electron microscopy images of the electrodes: (a) cross section of the TNT/Ti mesh after anodisation and (b) top view of Au@TNT/Ti. The inset in (a) shows the top view of nanotube arrays at high magnification.

SEM images of the TNT on Ti mesh (TNT/Ti), also modified with gold (Au@TNT/Ti), are reported in Fig. 1a and b, respectively. The SEM cross section shown in Fig. 1a clearly reveals the nanotubular structure (mesoporosity) of the TNT (average length of 1.5 μm) with an inner diameter in the range of 60–80 nm (see the inset in the figure).

A high dispersion of Au nanoparticles (range 20–80 nm) is also evidenced in the SEM images of Au@TNT/Ti reported in Fig. 1b. The EDX analysis in Fig. S2† reveals a Au weight concentration of 3.8% on the surface.

The XRD patterns, shown in Fig. S3,† confirm the presence of the anatase phase of TiO_2_ in both electrodes obtained after the crystallisation treatment of the anodised Ti mesh. The anatase crystalline phase (with peaks at 24.8° (101), 37.3° (103), 47.6° (200), 53.5° (105), and 55.1° (211), JCPDS card no. 21-1272) is the most desired one for photocatalytic applications, owing to its indirect bandgap and greater oxidative capacity. The presence of metallic titanium is also evidenced (peaks at 35° (100), 38° (002), 40.3° (101), and 53.1° (102), JCPDS card no. 44-1294). No gold-related peaks were detected. This is probably due to the low concentration and high metal dispersion.

The effect of the gold nanoparticles is evidenced in the UV-visible reflectance spectra of the electrodes (see Fig. S4†). Both are characterised by the presence of a peak in the UV region, typical of TiO_2_ (below 400 nm), and a broad band in the visible region owing to the metallic reflection of the Ti central core of the mesh and the defects of the nanotube structure (red part).^56^ The presence of the gold nanoparticles leads to a reduction in the UV region band, as a result of a shielding effect of the metals on the surface^57^ and in the red part of the visible region because of the loading of Au on superficial defect sites. An increase in the absorbance in the 400 and 500 nm range for the Au@TNT/Ti sample indicates the typical SPR effect of the gold nanoparticles.^58^

**Fig. 2:**
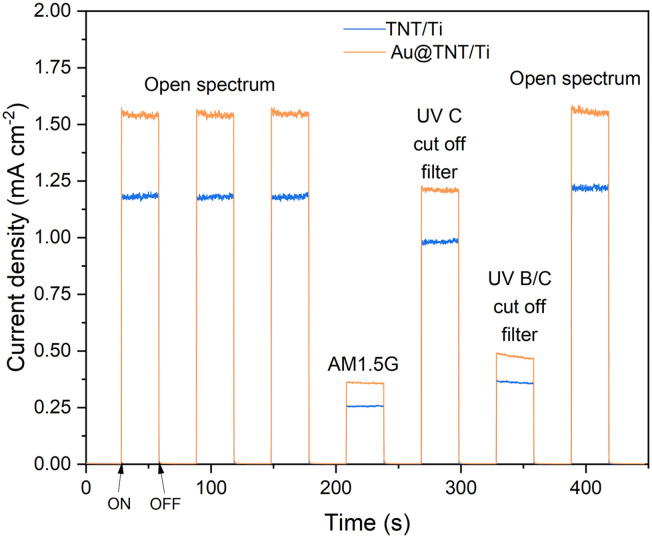
Chronoamperometric measurements for the TNT/Ti mesh (in blue) and the Au@TNT/Ti mesh (in orange) (+1.136 V *vs.* RHE, KOH 1 M electrolyte) under an open spectrum and with the application of filters (AM1.5G, UVC cut-off filter, UV B/C cut-off filter); the light was switched on and off every 30 s.

Chronoamperometric measurements shown in Fig. 2, evidence an increase in the photocurrent density related to the presence of the gold nanoparticles under all the lighting conditions. The greater response under the open spectrum indicates that Au loading facilitates the separation of charge carriers by providing an electron sink, as reported in the literature.^27,59^ Under simulated sunlight (with low UV radiation), the photo response of Au-TNT/Ti shows a significant enhancement compared with that of bare TNT. This effect may indicate an enhanced absorption in the visible range as a consequence of (i) the SPR effect of Au nanoparticles and (ii) the Schottky barrier formation at the interface between the metal and the semiconductor, which reduces the electron–hole recombination,^60,61^ as also confirmed by UV-vis spectra.

### Effect of power on performances of the TNT/Ti mesh

**Fig. 3:**
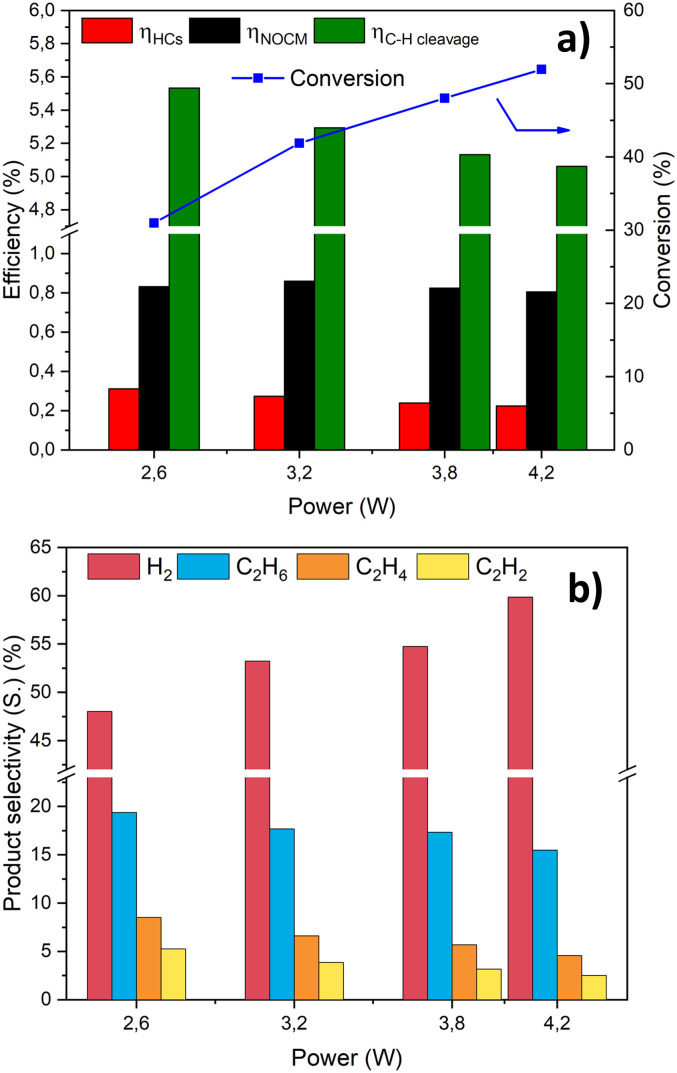
Effect of power on (a) efficiency and conversion and (b) product selectivity to hydrogen, ethane, ethylene and acetylene. Experimental conditions: frequency 22 kHz; no gap; feed flow 10 mL min^−1^.

A schematic illustration of the novel planar reactor integrating the photocatalytic TNT on the Ti mesh, together with a scheme of the setup for plasma catalytic testing and measurements is presented in the ESI.† Firstly, the effect of increasing power on efficiency, conversion and selectivity in plasma-only tests using the TNT/Ti mesh catalyst was studied, and the results are presented in Fig. 3.

The results obtained with TiO_2_/Ti (*e.g.* TiO_2_ anatase on Ti mesh) and Ti mesh alone compared to TNT/Ti (at similar power) are reported in the ESI† (Fig. S5), confirming the fundamental role of the nanoarchitecture (TNT) in promoting plasma–catalyst interaction.

Fig. 3a shows the correlated increase between conversion and plasma power. Specifically, Fig. 3a reports three distinct measures of efficiency: (i) energy converted into hydrocarbon products (red bar), (ii) energy converted into chemical energy (hydrocarbon + coke, where the latter accounts for the missing carbon balance) (black bar) and (iii) energy used for the cleavage of C–H bonds (green bar) (the details of calculations are reported in the ESI†).

The difference between the red and black bars indicates the energy amount transformed into solid carbon-based products. The green bar represents the amount of power being used efficiently, while the remaining fraction represents “wasted” energy, either lost as heat^25^ or due to back reactions.^62^

The observed low-efficiency values are typical or even slightly better than those reported for DBD processes,^13,63,64^ particularly at high conversion rates. As the conversion increases, there is a corresponding increase in the energy lost, due to collisions of vibrationally excited species and back reactions.^25^

A significant challenge of this reaction lies in the controlled C–H activation. Since the methane coupling is only slightly endothermic, achieving higher efficiencies becomes complex due to the energy released during the downhill part of the reaction pathway.

To properly analyse this point, an alternative process to the non-oxidative coupling of methane to C_2+_ hydrocarbons, should be introduced: the dry (oxidative) reforming of methane with CO_2_ to produce syngas. In dry reforming, CO_2_ reduction represents a way to store electrical energy into chemical energy^65^ thanks to the low chemical potential of CO_2_. Instead, converting methane into higher hydrocarbons is impeded by significant bond activation energy requirements. On the other hand, the dry reforming is highly endothermic, and the syngas needs to be further converted. Thus, methane non-oxidative coupling has different aims than dry reforming.

The NOCM is not primarily intended for energy storage but for producing high-value chemicals. Consequently, when evaluating performance factors, it is crucial to prioritise production costs, rather than energy efficiency.

**Fig. 4:**
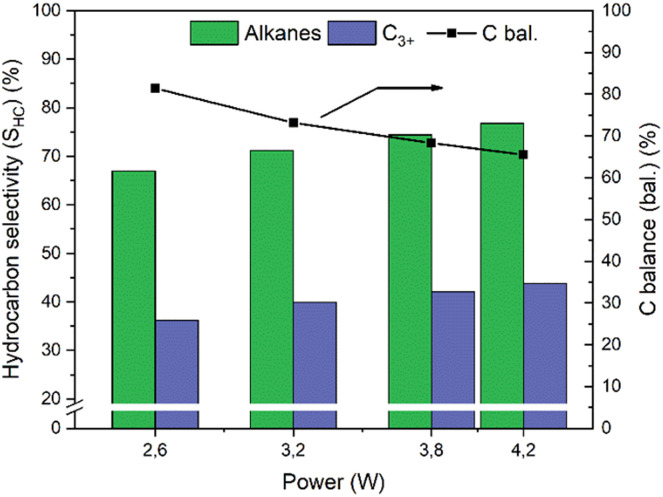
Effect of power on hydrocarbon selectivity and carbon balance. Experimental conditions as in Fig. 3.

In our case, the results are reported as a function of the power, which is directly proportional to the specific energy input (SEI, kJ L^−1^). While the efficiencies towards the formation of coupling products (*η*_NOCM_ and *η*_HCs_) remain rather constant as the power increases, the efficiency for breaking the C–H bonds decreases due to the back reactions, specifically the re-hydrogenation of excited CH_*x*_ species to methane^66^ (see Fig. 3a). As the conversion increases, more hydrogen and H-based excited species are generated (see Fig. 3b). The hydrogenation reactions become more probable and thus favoured.^67^ Indeed, the hydrogen selectivity rises to relatively high values. On the other hand, a decrease in all C_2_ products is observed as the power increases. This can be explained by an increase in coupling reactions that produce more C_3+_ products, as evidenced by the hydrocarbon selectivity trend shown in Fig. 4, which reports the selectivity towards alkanes and C_3+_ products.

The results reported in Fig. 4 indicate two key aspects: first, an increase in power enhances alkane selectivity due to the back reactions represented by eqn (1):

1 C_*x*_H_*y*_ + H (or H_2_) → C_*x*_H_*y*+*z*_

This phenomenon is induced by the shift in equilibrium towards re-hydrogenation, according to the Le Châtelier principle.^67^ Second, as the power intensifies, more C–C bonds are formed. This is related to the stronger electric field that produces more dehydrogenated reactive species (such as CH^*^_2_, CH^*^, or C^*^), which are more prone to form C_3+_ when compared to CH^*^_3_ active species, which primarily yield ethane.^68^ Moreover, as the power rises, a higher probability of coupling CH_*x*_ species to already formed C_2_H_*y*_ species also increases the yield of C_3+_ products.^69^ The former aspect (which involves a higher concentration of dehydrogenated excited species) increases the likelihood of the total dehydrogenation of methane into coke, thereby reducing the carbon balance.^70^ In addition, the heavier the hydrocarbons produced, the higher the probability of polymerisation into condensed carbonaceous-type C_*x*_H_*y*_ phases.^71^

### Effect of gold loading

**Fig. 5:**
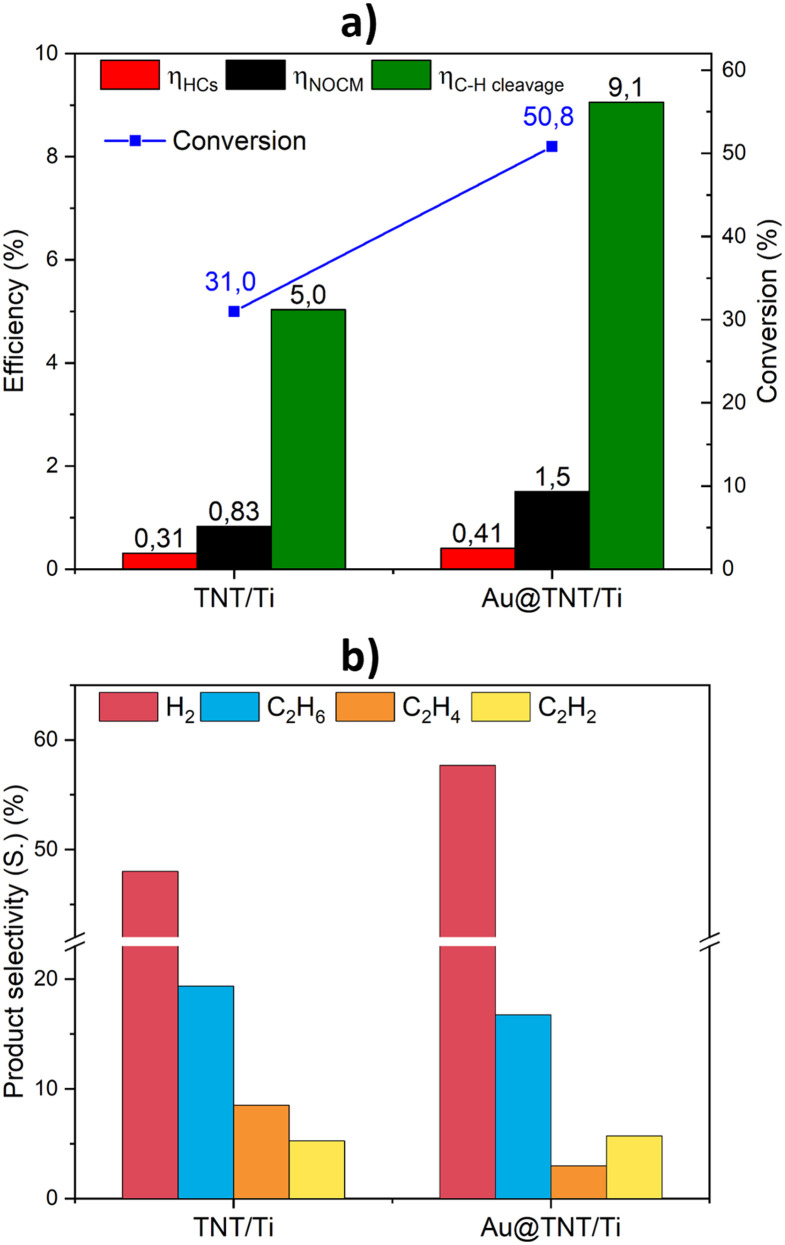
Effect of Au loading over the TNT/Ti mesh on (a) efficiency and conversion and (b) product selectivity to hydrogen, ethane, ethylene and acetylene. Experimental conditions: frequency 22 kHz; no gap; power 2.6 W (SEI = 156 kJ L^−1^) for the bare support and 2.28 W (SEI = 137 kJ L^−1^) for the Au-loaded support; feed flow 10 mL min^−1^.

Tests at similar power (around 2.3–2.6 W) were made to investigate the effect of Au addition to the TNT on Ti mesh support (Au@TNT/Ti). The gold loading considerably influences the performances, as shown in Fig. 5.

**Fig. 6:**
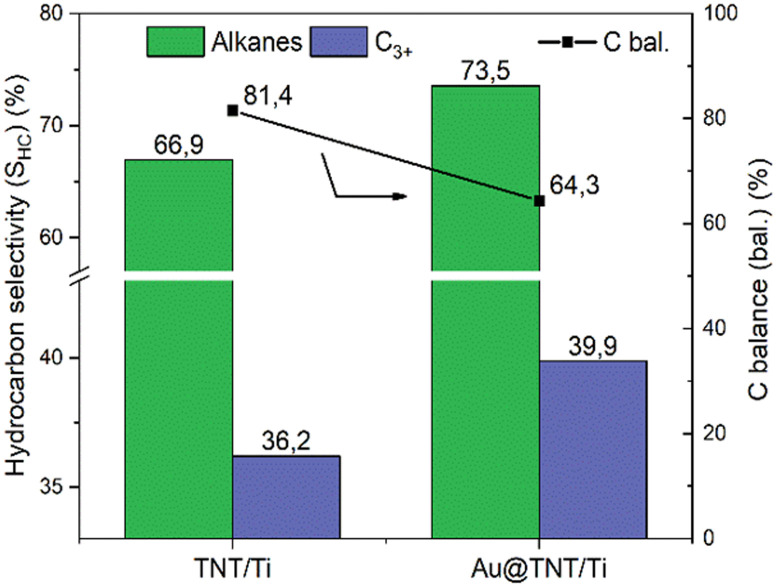
Effect of Au loading over the TNT/Ti mesh on hydrocarbon selectivity and carbon balance. Experimental conditions as in Fig. 5.

First, all efficiencies are significantly boosted, with a substantial rise of 64% in conversion with the Au-modified sample compared to the bare support (Fig. 5a). The benefits of adding a co-catalyst are evident from these results, but further optimisation of the co-catalyst nature and loading can improve the performances.^72^ Analysis of the selectivity values reported in Fig. 5b, indicates that the Au-loaded sample exhibits a higher selectivity towards hydrogen while the ethane selectivity decreases. In addition, the shift in selectivity between ethylene and acetylene must be noted. For the Au-loaded sample, the acetylene selectivity surpasses that of ethylene, which is the opposite trend observed for the bare support. Furthermore, an increase in selectivity towards alkanes and C_3+_ hydrocarbons is observed using the Au-loaded sample, as shown in Fig. 6.

The observed promotion in acetylene formation, compared to ethylene, with the Au-modified sample, could be ascribed to the dehydrogenation properties of Au nanoparticles.^73^ Furthermore, the decrease in carbon balance may also suggest the dehydrogenation properties of Au.^74^ On the other hand, the enhanced selectivity towards alkanes and C_3+_ hydrocarbons may indicate a hydrogenation behaviour for gold, in addition to the effect related to the higher methane conversion, as commented before. The apparent contradiction in these results likely indicates that the main role of Au nanoparticles (NPs) (in the plasma-only experiments) is not associated with their possible catalytic activity (tests are made at room temperature, where the Au@TNT/Ti catalyst is largely inactive in the indicated hydrogenation/dehydrogenation reactions). It is important to emphasise that the voltage–current traces for TNT/Ti and Au@TNT/Ti are nearly identical (see Fig. S6a and b†), allowing us to exclude the electrical contribution to the superior results obtained with the Au-loaded catalyst.

The role of Au NPs is thus primarily associated with a change in the characteristics of plasma discharges.

The presence of Au NPs leads to a localised accumulation of charges when plasma species interact with the catalyst. Compared to crystalline TiO_2_, nanotubes exhibit a greater number of defects due to their unique growth mechanism.^75^ Au NPs preferentially localise at these defect sites, enhancing charge trapping at the interface. This accumulation of charges generates a local electrical field, which influences the micro-discharge characteristics.^76^ There is, thus, a physical mechanism underlying the micro-discharges in the plasma specifically associated with the deposition of Au NPs on the TNT/Ti mesh. This may explain the observed change in the activity as well as the modification in selectivity and energy efficiency.

On the other hand, the interface of Au NPs with defective TNT sites would generate polaron charge trapping,^77^ leading to an enhanced exciton polaron in titania that could enhance the coupling with the excited molecules of the plasma, thus influencing the paths of transformation and selectivity.^78^ The two effects are highly interconnected. We believe that these effects are both responsible for the drastic change in the behaviour of TNT/Ti and Au@TNT/Ti catalysts.

### Effect of light irradiation

**Fig. 7:**
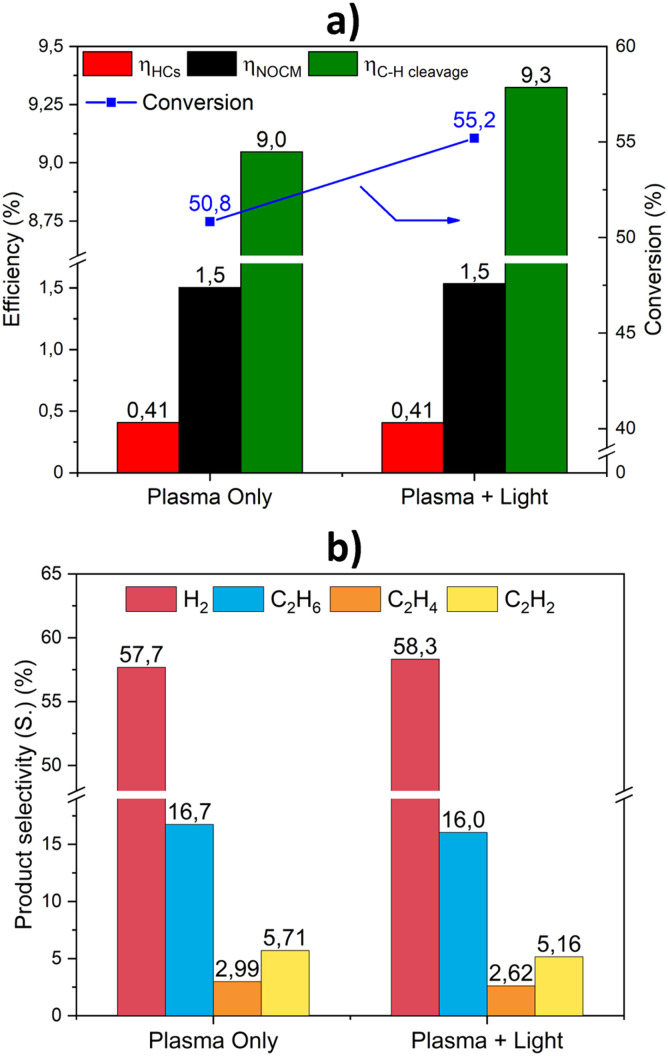
Effect of light irradiation in assisting plasma conversion on (a) efficiency and conversion and (b) selectivity to hydrogen, ethane, ethylene and acetylene for Au@TNT/Ti. Experimental conditions: frequency 22 kHz; no gap; power 2.28 W (SEI = 137 kJ L^−1^) for plasma and 2.38 W (SEI = 143 kJ L^−1^) for light–plasma reaction; feed flow 10 mL min^−1^; irradiation 250 mW cm^−2^, with an open spectrum Xe lamp (300 W).

**Fig. 8:**
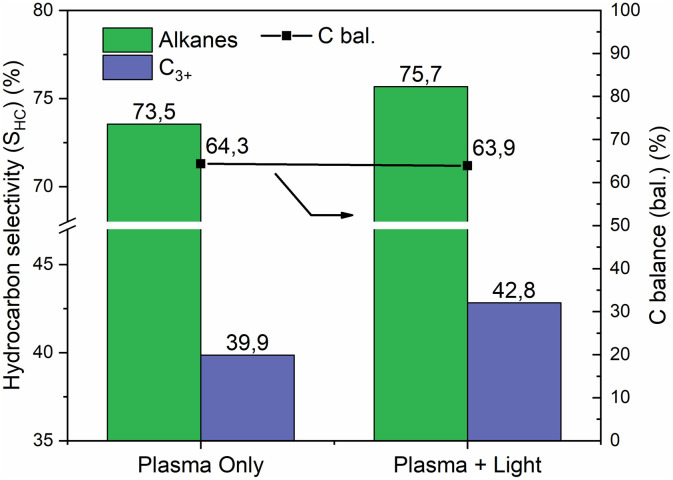
Effect of light irradiation in assisting plasma conversion on hydrocarbon selectivity and carbon balance for Au@TNT/Ti. Experimental conditions as in Fig. 7.

After tests in the plasma-only conditions, the effect of light irradiation and its synergy with plasma were examined. The effects of assisting plasma conversion with light irradiation are presented in Fig. 7 and 8 for the Au@TNT/Ti sample, *e.g.* the most active one. It should be remarked that the results presented have been obtained at equal voltages and very similar plasma powers to be well comparable, and thermal contributions have also been excluded by measuring the temperature in the plasma zone. It should also be noted that without plasma, *e.g.* only by illuminating the sample, no conversion has been detected.

The main observed effect upon irradiation is an increase in methane conversion (Fig. 7a). While the reaction efficiencies remain similar, the C–H efficiency shows an improvement. Light irradiation synergistically interacts with plasma, increasing productivity and altering the pathways of transformation.

The trend in product selectivity, presented in Fig. 7b, indicates a higher amount of hydrogen produced, while a slight decrease in each C_2_ product is observed upon irradiation. The distribution between ethane, ethylene, and acetylene is nearly independent of whether the reactor is irradiated.

The analysis of the hydrocarbon selectivity (Fig. 8) highlights the impact of assisting the plasma methane conversion with light, evidencing an increase in the selectivity towards alkanes and C_3+_ upon irradiation. These results suggest that the photocatalytic contribution primarily facilitates the formation of more C–C bonds, rather than promoting dehydrogenation reactions.

This effect may indicate the photocatalytic generation of methyl radicals, as commonly reported in the literature.^19^

However, as reported above, under our conditions, there is almost no photocatalytic activity in the absence of plasma. It is therefore not reasonable that light irradiation solely enhances the formation of methyl radicals, as their coupling leads to species characterised by C–C bond formation.

**Fig. 9:**
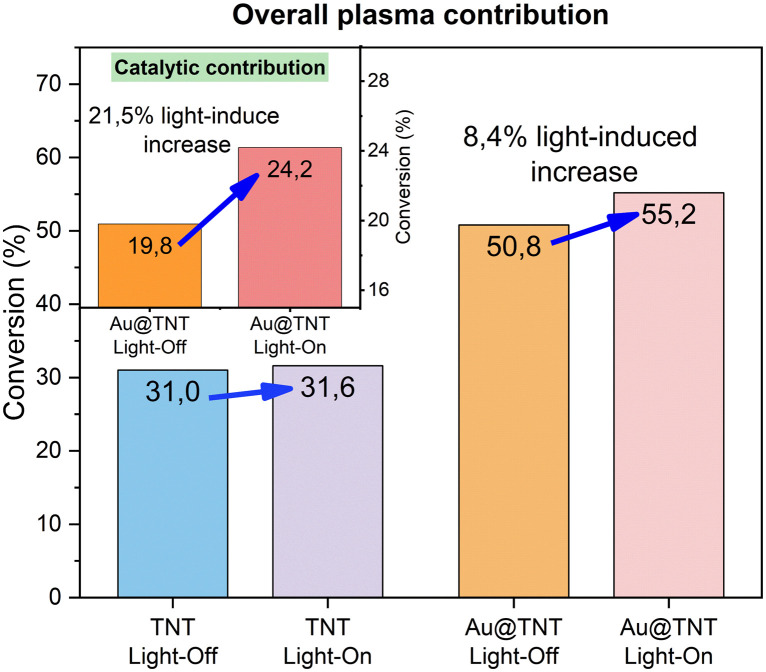
Effect of light irradiation on methane conversion for Au@TNT/Ti. Data for bare TNT/Ti are reported for comparison. In the inset, the sole catalytic surface contribution is reported. Experimental conditions as in Fig. 7.

Since plasma is a 3D (volumetric) process, while photocatalysis is mainly a 2D process (connected to the irradiated surface), it is necessary to consider this difference when determining the specific role of irradiation in the overall plasma reaction. Fig. 9 reports the increase in the conversion for Au@TNT/Ti with or without illumination (related to both 2D and 3D contributions), while the inset illustrates the exclusive catalytic surface contribution (calculated by subtracting the TNT 3D plasma contribution). The results for TNT/Ti without Au NPs are also given for comparison. With these assumptions, we could estimate that the light irradiation promotes the behaviour of Au@TNT/Ti, already significantly better than that of TNT/Ti, increasing by 21.5% the intrinsic rate of the NOCM surface process. This is the first time, to our knowledge, that this synergy could be demonstrated. The interpretation of this synergy is not related to the effect of generating charge separation that triggered the photocatalytic NOCM reaction, commonly reported in various studies, as cited before. In that case, the reaction rates are lower, or the amount of photocatalytic material is much higher. Under our conditions, thus, this typical photocatalytic behaviour does not account for enhanced performances. On the other hand, tests for the bare TNT/Ti in combination with light do not lead to a promotion in the behaviour, as shown in Fig. 9.

This synergy is thus specifically observed for Au@TNT/Ti samples, which already show a higher promotion of performance than bare TNT/Ti. There is a link between the Au NPs effect in promoting plasma-only activity (Fig. 5) and the effect of light (Fig. 7 and 8).

We thus assert that the most obvious interpretations – namely, that when photoelectrons accumulate on Au, the positively charged plasma sheath^79^ provides a pathway for the fast utilisation of photogenerated electrons, or the charge separation induced by light absorption creates additional centres of NOCM photoelectrons – are not correct, as previously commented.

The data highlight a more complex and still largely unexplored motivation based on two interconnected reasons: (i) the induced physical change in the nature of the plasma micro-discharges and (ii) the creation of specific surface vibrational states on the catalyst (deriving from the coupling between trapped surface charges at the gold/titania defects interface), along with localised phonons that positively interact with vibrationally excited species generated by the plasma,^79,80^ leading to enhanced performances.

A better understanding of these possibilities is the key to designing more effective systems and reactors that facilitate synergistic interaction between plasma and light in the NOCM reaction.

## Experimental

### Materials and methods

A Phenom ProX scanning electron microscope (SEM) equipped with EDS was employed to analyse the surface morphology of the electrodes.

Powder X-ray diffraction (XRD) patterns were recorded using a Bruker D2 Phaser diffractometer with a Ni β-filtered Cu-Kα radiation source. The diffractograms were obtained in a 2*θ* range going from 20° to 60°, with a scanning rate of 0.025° s^−1^.

The optical properties of the electrodes were evaluated by recording UV-vis diffuse reflectance spectra in the spectral range of 200–700 nm using a Thermo Fischer Evolution (220) spectrometer with an integrating sphere for solid samples.

Photocurrent profiles of the electrodes were recorded in a three-electrode cell using a potentiostat/galvanostat (Autolab PGSTAT204) at +0.1 V against an Ag/AgCl reference electrode (+1.136 V *vs.* RHE) (Amel) in 1 M KOH, under a continuous flow of N_2_ (5 mL min^−1^). A Pt wire was used as the counter-electrode. The working electrode was irradiated within an area of about 1 cm^2^ with a Lot-Oriel 300 W Xe arc lamp, with cycles of 30 s of illumination, followed by 30 s of dark, using different filters to cut out chosen wavelength ranges: the AM 1.5 G (standard solar irradiation on Earth) filter, UV C and UV B/C blocking filters.

The potential values referred to the Ag/AgCl electrode are translated to RHE using the following formula:*E*_(RHE)_ = *E*_(Ag/AgCl)_ + 0.059 pH + 0.21

### Procedures of synthesis

All commercially available compounds were purchased from Sigma-Aldrich and used without further purification.

### Synthesis of TiO_2_ nanotubes on the Ti mesh (TNT/Ti)

TiO_2_ nanotube arrays have been synthesised on a titanium mesh substrate consisting of 80 mesh woven from a 0.13 mm diameter wire, provided by Alfa Aesar. This synthesis was achieved through a controlled anodic oxidation process.^56^ The anodisation parameters have already been optimised for our purpose. Before anodisation, the samples were cleaned by ultrasonication in isopropyl alcohol for 30 min to eliminate any organic impurities and then dried naturally in air. Subsequently, the clean substrate, serving as the working electrode, was positioned against a Pt counter-electrode inside a two-electrode electrochemical cell made of Teflon. A potentiostat (Agilent E3612A) and a multimeter (Keithley 2000) were employed to maintain a constant potential of 50 V between the two electrodes for 1 hour while recording the current density. The electrolyte bath was prepared with 2.0 wt% distilled H_2_O and 0.3 wt% ammonium fluoride in ethylene glycol. After the anodisation, the samples were annealed at 450 °C for 3 hours, with a heating and cooling rate of 2 °C min^−1^, to induce crystallisation of the amorphous oxide nanotube arrays.

### Synthesis of the TiO_2_ layer on the Ti mesh (TiO_2_/Ti)

The anodisation of the Ti mesh to develop a TiO_2_ compact layer (TiO_2_/Ti) was conducted on the Ti mesh support with the same protocol as reported above, except for the electrolyte bath, which was composed of a 0.5 M Na_2_SO_4_ aqueous solution.

### Deposition of gold nanoparticles (Au@TNT/Ti)

Gold was loaded on the support by electrodeposition methodology. The TNT/Ti mesh, acting as a working electrode, was placed in a two-electrode cell made of Teflon with a standard Pt counter-electrode. A solution of HAuCl_4_·3H_2_O, 0.1 mM in deionised (DI) water, was used as the electrolyte at room temperature. The electrodeposition was carried out by applying a potential of −70 V with an Agilent potentiostat (Agilent E3612A) for 45 min.^44,81^

### DBD reactor and testing setup

To study the coupling between photocatalysis and non-thermal plasma, a specific dielectric barrier discharge (DBD) reactor with a planar geometry was designed and built. All details are reported in the ESI† (Fig. S7). The whole setup for plasma catalytic testing is illustrated in Fig. S8.† For the testing, the reactor was de-aired for 30 min; subsequently, a mix of 10% CH_4_, bal. Ar was flown at 10 mL min^−1^ for 1 hour to reach equilibrium. The use of auxiliary gases in plasma catalysis is usually reported as beneficial for methane conversion.^82,83^ We decided to conduct the experiments by adding Ar to the feed gas, to avoid issues related to C deposition and increase the electron temperature while promoting methane conversion through inelastic collisions and the Penning ionisation mechanism.^82^

A high voltage was applied at 22.0 kHz, and the power was adjusted by tuning the DC voltage. The power was calculated by the product of current and voltage measured by a Rogowski coil and an HV probe, respectively. For photo-assisted plasma conversion, a Xe lamp (300 W) (Quantum Design) was used for the irradiation of the reactor; a shutter was used to initiate reactor illumination simultaneously with plasma ignition. The gas flow rate in the outlet during the reaction was assessed using a soap-film flow meter. The products were quantified using a gas chromatograph (Agilent GC 8890, Ar/He carriers) equipped with Rt®-Q-Bond columns and flame ionization detectors (FIDs) for the analysis of hydrocarbons, and Poropak-Q and MS-13X columns and a thermal conductivity detector (TCD) to quantify hydrogen and check that there are no traces of O_2_, CO or CO_2_. All the experiments were carried out following the same protocol to ensure high reproducibility.

## Conclusions

A planar dielectric barrier discharge (DBD) reactor featuring a quartz window for catalyst irradiation was designed and tested using a photoactive TNT/Ti mesh that acts as both a catalyst and an electrode. This mesh was further modified with Au nanoparticles to boost its light absorption, charge carrier separation and reactivity.

The effects of applied power on conversion, efficiency, selectivity and carbon balances have been investigated. The introduction of gold significantly enhanced the performance, resulting in a substantial rise of 64% (SEI = 137 kJ L^−1^) in the conversion rate compared to the TNT/Ti bare support, accompanied by a shift in selectivity, pushing towards the production of alkanes and C_3+_.

For the first time, an effective synergistic mechanism between plasma and light has been proven for gold-modified materials. Light irradiation enhanced the behaviour of Au@TNT/Ti, already significantly better than that of TNT/Ti, increasing by 21.5% the intrinsic rate of the NOCM surface process.

The discussion of the reasons for this synergic effect, however, pointed out that the interpretation connected to the effect of light absorption in creating additional centres of the NOCM is incorrect. The data highlight a more complex and still largely unexplored motivation based on two interconnected reasons: (i) the induced physical change in the nature of the plasma micro-discharges and (ii) the creation of specific surface vibrational states in the catalyst, which positively couple with the vibrationally excited species generated by the plasma. Although further studies are needed for a deeper insight into these factors to optimise the process, even in a future industrial perspective, the obtained results open new possibilities for exploiting the synergistic interaction of plasma and light, especially in a challenging reaction such as the NOCM.

## Author contributions

VL: investigation, data curation, formal analysis, methodology, conceptualization, writing – original draft. LdP: investigation, data curation. SP: project administration. GC: validation, writing – review & editing, project administration, funding acquisition. CG: methodology, conceptualization, validation, supervision, writing – review & editing.

## Conflicts of interest

There are no conflicts to declare.

## Supplementary Material

CY-015-D5CY00206K-s001

## Data Availability

Data are available upon request from the corresponding author.
