# Supplementary material for: Synergistic effects of light and plasma catalysis on Au-modified TiO2 nanotube arrays for enhanced non-oxidative coupling of methane
Source: Catal Sci Technol. 2025 May 1;15(12):3725–35. doi: 10.1039/d5cy00206k (PMC12089975; doi:10.1039/d5cy00206k)
Supplement: CY-015-D5CY00206K-s001 [file CY-015-D5CY00206K-s001.pdf]

Supplementary Information (SI) for Catalysis Science & Technology.
This journal is © The Royal Society of Chemistry 2025

Supporting Information

Synergistic effects of light and plasma catalysis on Au-modified TiO_2 nanotube arrays for enhanced non-oxidative coupling of methane

Victor Longo, Luana De Pasquale, Siglinda Perathoner, Gabriele Centi and Chiara Genovese*

Department of Chemical, Biological, Pharmaceutical and Environmental Sciences (ChiBioFarAm),
University of Messina, ERIC aisbl and CASPE/INSTM
Viale Ferdinando Stagno d'Alcontres 31, 98166 Messina (Italy)

*chiara.genovese@unime.it

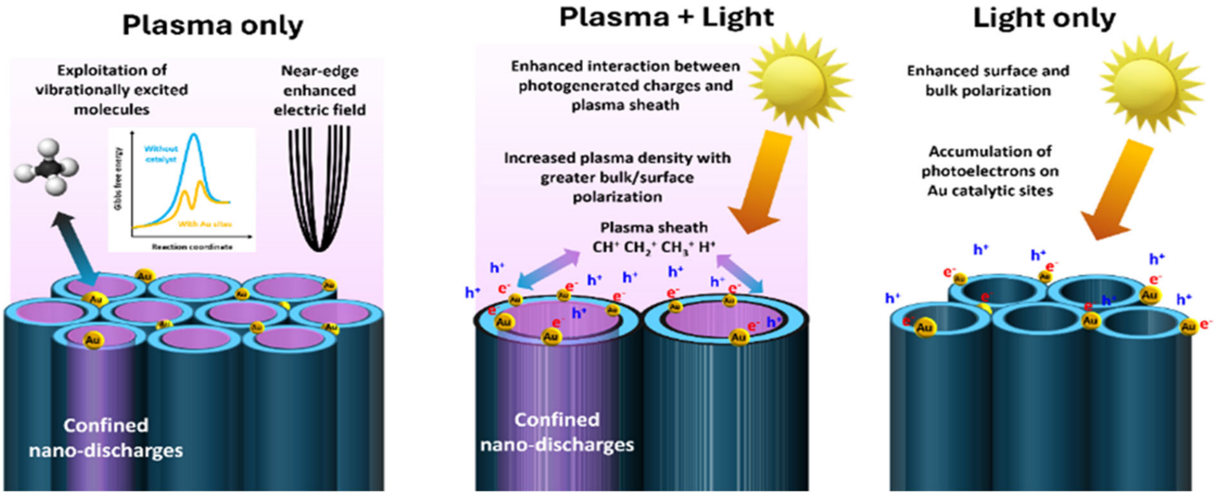

Figure S1. Scheme of the advantages of Au-TiO_2 nanotube utilisation in plasma catalysis. The structure of the nanotubes provides an enhanced electric field near the edge of the tubes, offering a path for plasma diffusion into the pores, where the vibrationally excited molecules can then be activated over catalytic species (Au nanoparticles) loaded into the walls

Supplementary Results

SEM-EDX analysis

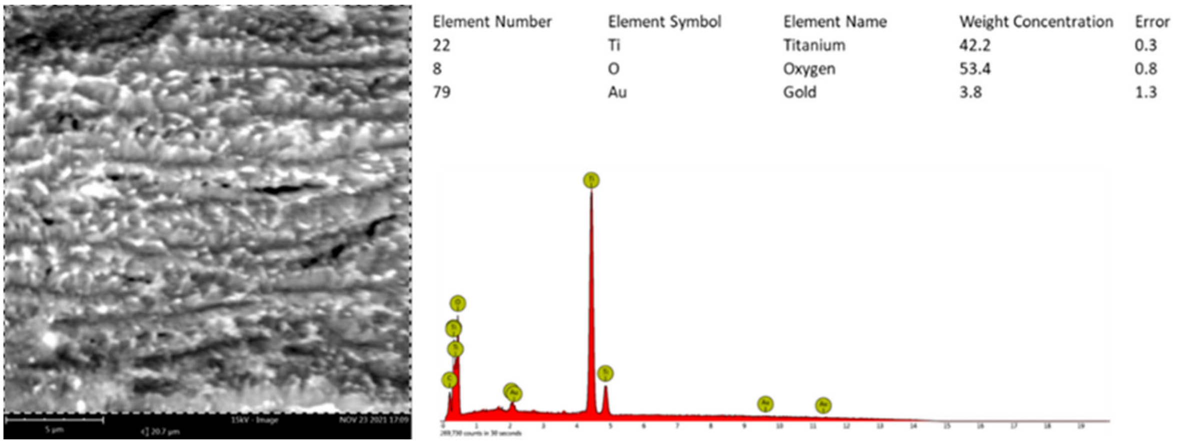

Element Number	Element Symbol	Element Name	Weight Concentration	Error
22	Ti	Titanium	42.2	0.3
8	O	Oxygen	53.4	0.8
79	Au	Gold	3.8	1.3

Figure S2. SEM-EDX analysis of Au@TNT/Ti mesh

XRD analysis

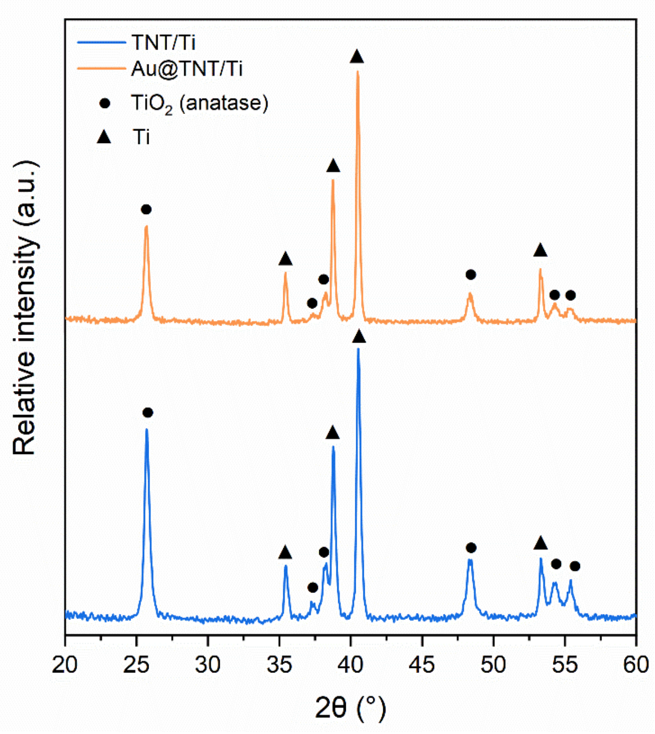

Figure S3.1 XRD patterns of the TNT/Ti mesh (in blue) and Au-modified TNT/Ti mesh (in orange). The peaks related to the titanium and the anatase phase of TiO_2 are marked with a triangle and a circle.

UV-Vis diffuse reflectance

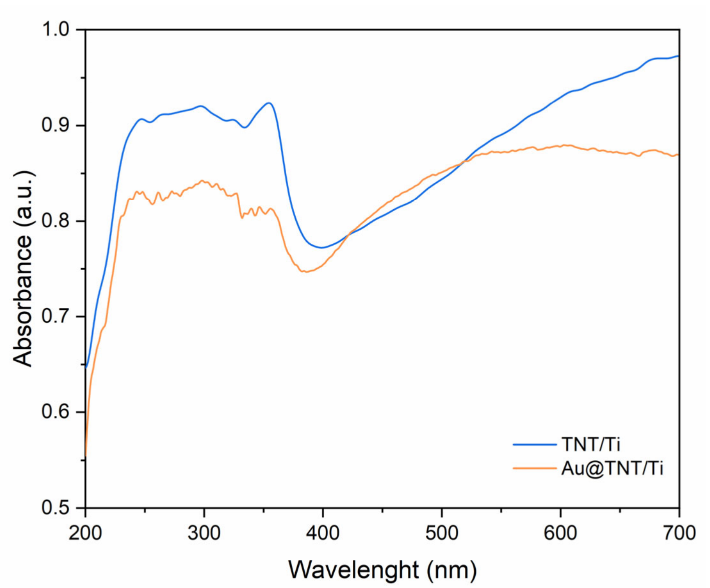

Figure S4. UV-Vis diffuse reflectance spectra of TNT/Ti mesh (in blue) and Au-modified TNT/Ti mesh (in orange).

Plasma catalytic tests

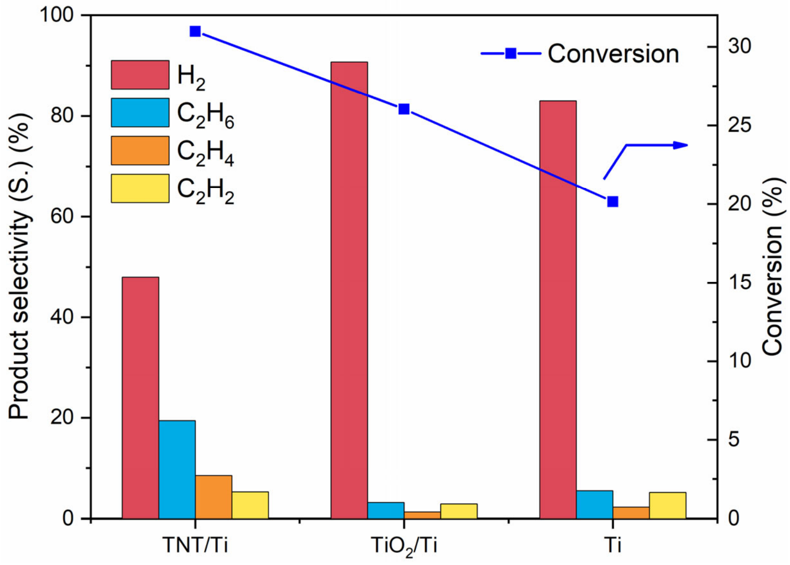

Figure S5. Conversion and product selectivity to hydrogen, ethane, ethylene and acetylene for TNT/Ti mesh, TiO_2/Ti (e.g. TiO_2 anatase on Ti mesh) and Ti mesh alone. Experimental conditions: frequency 22kHz; no gap; power 2.6W (SEI = 156 kJ·L^{-1}) for TNT/Ti and 2.2 W (133 kJ·L^{-1}) for TiO_2/Ti and Ti mesh alone, feed flow 10 mL·min^{-1} (10% CH_4, balance Ar).

Voltage-current tests

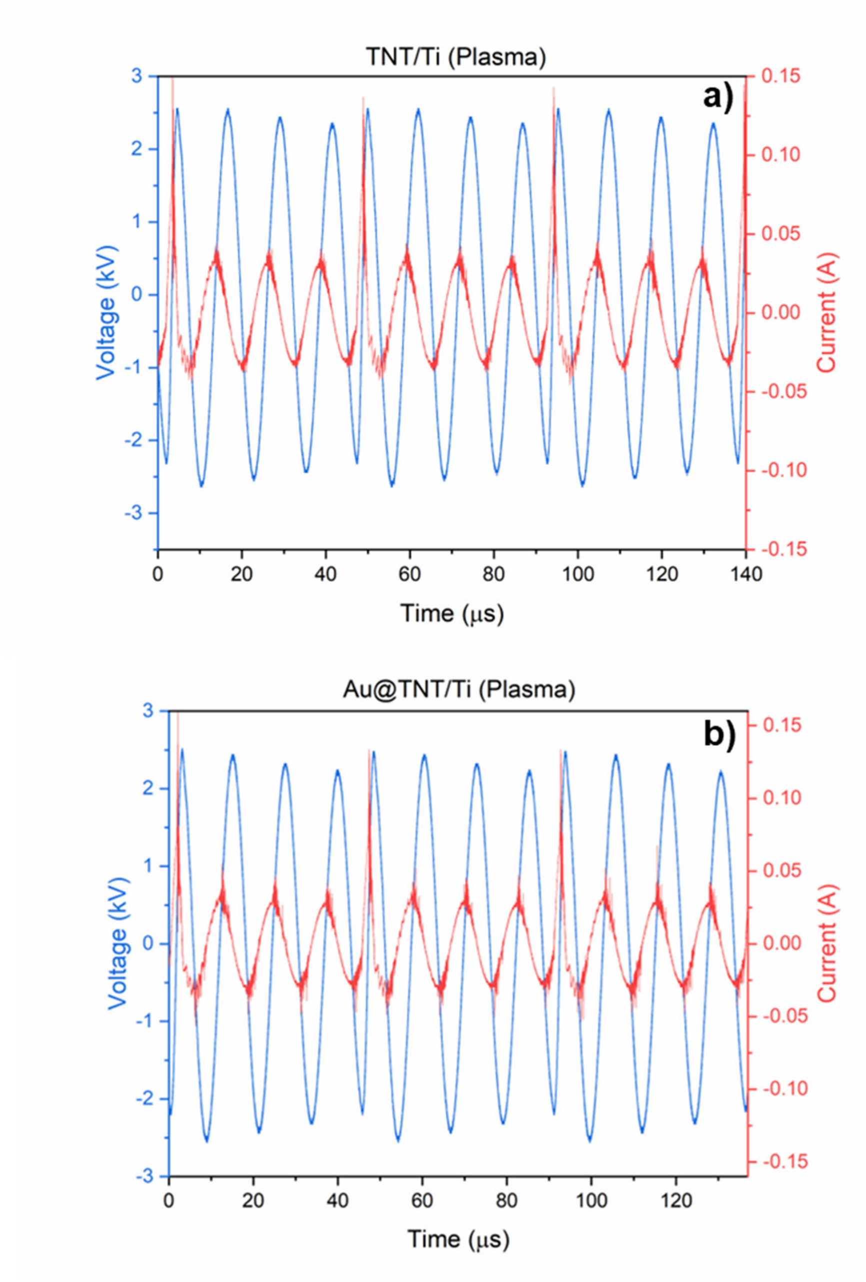

Figure S6. Voltage-current traces for the TNT/Ti sample(a) and Au@TNT/Ti sample (b) in dark conditions. The operating frequency is 22kHz.

Experimental

DBD reactor and setup

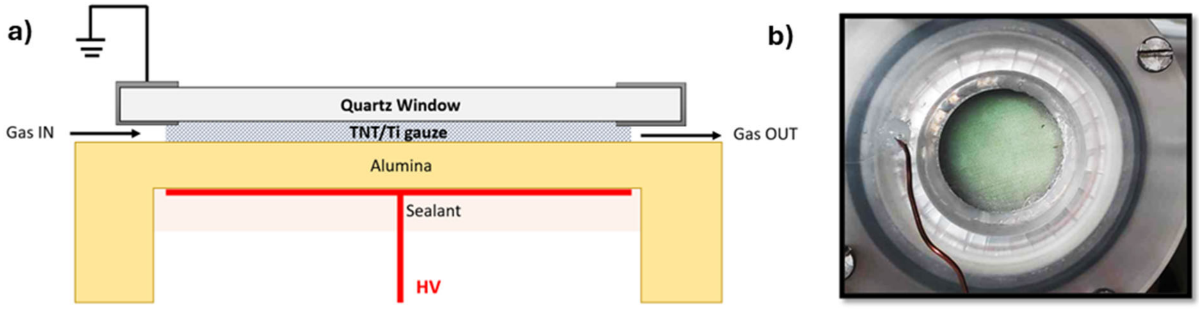

Figure S7. (a) Schematic illustration of the planar reactor integrating the photocatalytic TNT/Ti mesh and (b) pure methane plasma observed in the reactor.

The reactor consists of two parts: 1) a section made in Macor (machinable glass ceramic material) with a quartz window for the illumination of the catalyst (consenting to study the interaction between light and non-thermal plasma) 2) an alumina section (plasma zone) with a “cap-like” geometry to lengthen the electron path and prevent short circuits where the (photo)catalyst is placed. A stainless-steel disc acts as high voltage (HV), directly connected to the power supply, while the electrode (TNT/Ti mesh) is grounded. A radial gas entrance was adopted to flow the gas directly into the plasma chamber. The photocatalytic mesh (TNT/Ti mesh, 34 mm in diameter) is in direct contact with the dielectric barrier, i.e. the quartz window, to form surface plasma rather than volume plasma to maximize the fraction of gas/plasma at the catalyst surface interface. To investigate the effect of light irradiation, we conducted two separate plasma experiments—one in the dark and the other under light irradiation, for motivations related to plasma stabilization and analytical issues, but ensuring to operate in identical conditions. The whole setup for plasma catalytic testing is illustrated in Figure S8.

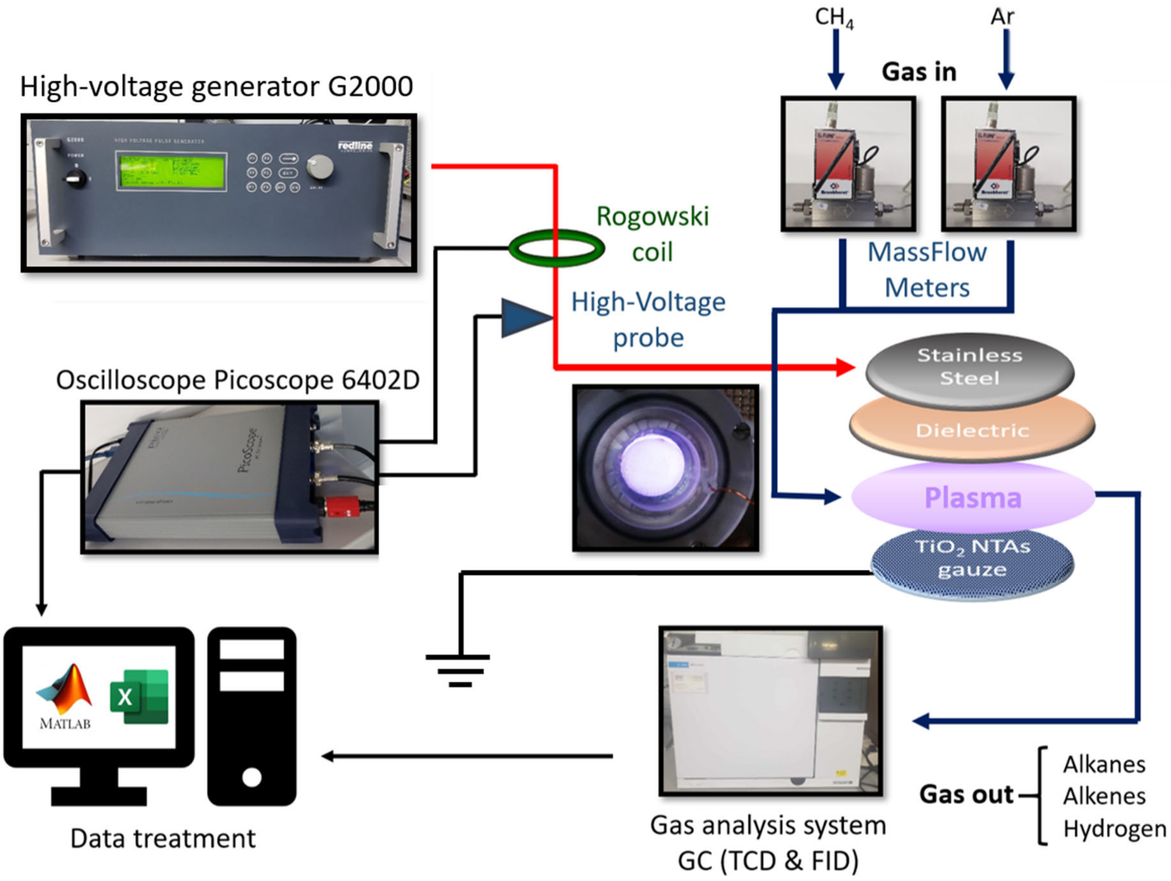

Figure S8. Scheme of the setup for plasma catalytic testing and measurements.

Specifically, mass flow meters (Bronkhorst) were used to flow the gases: Ar (grade 5.2) and CH_4 (grade 4.0, with less than 5 ppm H_2O and O_2). A G2000 (RedLine Technologies) generator was used to supply high voltage with a fixed resonance frequency, operating on the 4th harmonics (around 20 kHz). When operated on the harmonics of the resonance frequency, the output is a periodic train of damped sine waves with as many impulses as the harmonic number. The electrical signals were recorded with a digital oscilloscope (Picoscope 6402D). On the high-voltage side, a high-voltage probe (CalTec CT4028, voltage dividing ratio: 1:1000) and a Rogowski coil (Pearson 4100 current: voltage converting ratio 1A:1V) were used to measure the high voltage and the current, respectively. The plasma power was determined by the product of current and voltage. This methodology was preferred to the Lissajous method, because the G2000 power supply provides trains of damped sine waves, thus, the corresponding Lissajous figure does not correspond to a closed repeating figure[1,2]. A Faraday cage was used to prevent electromagnetic noise resulting from the high voltage.

Mathematical methods

The **conversion** has been determined according to $Eq. (1)$.

$$Conv. (\%) = \frac{[CH_4]_{IN} - [CH_4]_{OUT}}{[CH_4]_{IN}} \times 100\% \qquad Eq. (1)$$

With, $[CH_4]_{IN}$ and $[CH_4]_{OUT}$ the molar concentrations of methane measured before and during reaction, respectively.

The Specific Energy Input (SEI), expressed in kJ L^{-1} of CH_4 was determined according to $Eq. (2)$. The Specific Energy Requirement (SER), corresponding to a normalization of the SEI by the conversion, was established as in $Eq. (3)$.

$$SEI\ (kJ. L^{-1}) = \frac{Power\ (W)}{Flow_{(CH_4)}(mL\ min^{-1})} \times 60 \qquad Eq. (2)$$

$$SER(kJ. mol^{-1}) = \frac{SEI\ (kJ\ mol^{-1})}{Conv.} \times V_M \qquad Eq. (3)$$

with the conversion expressed as the fraction of mole converted, and V_M, the molar volume, equal to 24.45 L mol^{-1}.

The **energy efficiencies** were determined as in *Eq* (4), (5) and (6). The efficiency for C-H bond breaking ($\eta_{C-H\ cleavage}, Eq. (4)$), describes the amount of electrical energy used to break the C-H bond (hence only the uphill part of the reaction path), the efficiency for the formation of hydrocarbons ($\eta_{HCs}, Eq. (5)$), describes the energy converted into only hydrocarbons (without considering carbon deposition), and the efficiency of the global reaction ($\eta_{NOCM}, Eq. (6)$) describes the amount of electrical energy converted into chemical energy (related to the formation of gas products and coke).

$$\eta_{C-H\ cleavage}\ (\%) = \frac{\Delta H^0_{C-H\ cleavage}\ (kJ\ mol^{-1})}{SER\ (kJ\ mol^{-1})} \qquad Eq. (4)$$

$$\eta_{HCs}\ (\%) = \frac{\Delta H^0_{NOCM\ HCs}\ (kJ\ mol^{-1})}{SER\ (kJ\ mol^{-1})} \qquad Eq. (5)$$

$$\eta_{NOCM}\ (\%) = \frac{\Delta H^0_{NOCM}\ (kJ\ mol^{-1})}{SER\ (kJ\ mol^{-1})} \qquad Eq. (6)$$

With $\Delta H^0_{C-H\ cleavage}$ corresponding to the change in enthalpy for breaking one mole of C-H bond from the methane molecule (434 kJ.mol^{-1}).

The global NOCM reaction can be described as the sum of all different hydrocarbon products (HC) of chemical formula (C_xH_y) weighted by a stoichiometric coefficient determined from product analysis (α), while the quantity of hydrogen and eventual carbon are determined by balancing C and H atoms, from hydrocarbon products, as in $Eq. (7)$.

$$CH_4 \rightarrow \sum_{i=1}^{i=21} \alpha_i \times C_xH_{y_i} + \left(1 - \sum_{i=1}^{i=21} x \times \alpha_{i(C_xH_y)}\right) C_{(s)} + \left(2 - \sum_{i=1}^{i=21} {}^{y}/_{2} \times \alpha_{i(C_xH_y)}\right) H_2 \qquad Eq. (7)$$

CH_4 represents the quantity of mole converted, normalized to 1, and i stands for each hydrocarbon products identified during the reaction (total of 21). The number of carbon and hydrogen atoms in a hydrocarbon product is defined by x and y, respectively.

$\Delta H^0_{NOCM\ HCs}$ and ΔH^0_{NOCM} correspond to the global enthalpy of reaction with the respective equation ($Eq.(8)$ and $Eq.(9)$:

$$\Delta H^0_{NOCM\ HCs} = \sum_{i=1}^{i=21} x \times \alpha_{i_{(C_xH_y)}} \times \Delta H^0_{r_{(C_xH_{y_i})}} \qquad Eq.(8)$$

$$\Delta H^0_{NOCM} = \Delta H^0_{NOCM\ HCs} + \left(1 - \sum_{i=1}^{i=21} x \times \alpha_{i_{(C_xH_y)}}\right) \times \Delta H^0_{r_{(CH_4 \rightarrow C_{(s)} + 2H_2)}} \qquad Eq.(9)$$

With the enthalpy of reactions (from methane coupling) of each products ($\Delta H^0_{r_{(HC_i)}}$) determined based on the enthalpy of formation of each product [65].

The **product selectivity**, $S_{Product}$, ($Eq.(10-11)$) (taking hydrogen and hydrocarbons into account) was established with:

$$S_{H_2}\ (\%) = \frac{0.5 \times [H_2]\ (mol)}{\sum_{i=1}^{i=21} y/4 \times C_xH_{y_i}\ (mol) + 0.5 \times [H_2]\ (mol)} \times 100\% \qquad Eq.(10)$$

$$S_{C_xH_y}\ (\%) = \frac{x \times [C_xH_y]\ (mol)}{\sum_{i=1}^{i=21} y/4 \times C_xH_{y_i}\ (mol) + 0.5 \times [H_2]\ (mol)} \times 100\% \qquad Eq.(11)$$

The **hydrocarbon selectivity** $S_{HC_{Product}}$, ($Eq.(12-15)$) (considering only hydrocarbon products) was established i) for the sum of saturated (alkanes, $S_{HC_{(Alkanes)}}$, $Eq.(12)$) and unsaturated (alkenes and acetylene, $S_{HC_{(Unsaturated)}}$, $Eq.(13)$) compounds (the sum of alkanes and unsaturated hydrocarbon selectivity = 100%), and ii) for the C_2 (ethane, ethylene and acetylene, $S_{HC_{(C2)}}$, $Eq.(14)$) and C_{3+} compounds ($S_{HC_{(C3+)}}$, $Eq.(15)$) (the sum of C_2 and C_{3+} hydrocarbon selectivity = 100%).

$$S_{HC_{(Alkanes)}}\ (\%) = \frac{\sum_{i=1}^{i=21} n \times [alkanes\ (C_nH_{2n+2})]\ (mol)}{\sum_{i=1}^{i=21} x \times [C_xH_y]_i\ (mol)} \times 100\% \qquad Eq.(12)$$

$$S_{HC_{(Unsaturated)}}\ (\%) = \frac{\sum_{i=1}^{i=21} n \times [alkene\ (C_nH_{2n})] + 2 \times [acetylene]\ (mol)}{\sum_{i=1}^{i=21} x \times [C_xH_y]_i\ (mol)} \times 100\% \qquad Eq.(13)$$

$$S_{HC_{(C2)}}\ (\%) = \frac{2 \times ([C_2H_6] + [C_2H_4] + [C_2H_2])\ (mol)}{\sum_{i=1}^{i=21} x \times [C_xH_y]_i\ (mol)} \times 100\% \qquad Eq.(14)$$

$$S_{HC_{(C3+)}}\ (\%) = \frac{3 \times [C_3] + 4 \times [C_4] + 5 \times [C_5] + 6 \times [C_6]\ (mol)}{\sum_{i=1}^{i=21} x \times [C_xH_y]_i\ (mol)} \times 100\% \qquad Eq.(15)$$

The **carbon balance (C_{bal})** was determined according to equations $Eq.(16)$.

$$C_{bal}(\%) = \frac{\sum_{i=1}^{i=21} x \times [C_xH_y]_i + [CH_4]_{OUT}}{[CH_4]_{IN}} \times 100\% \qquad Eq.(16)$$

The **plasma power ($Power$)** was determined according to equations $Eq.(17)$.

$$Power = f \times \int_0^T U_{HV} \times i_{Rog}\, dt \qquad Eq. (17)$$

With, f the frequency of a train of pulses (Hz), T the duration of a train of pulses (s), U_{HV} the high voltage measured by the HV probe (kV), and i_{Rog} the current measured via the Rogowski coil (mA).

References

1. R. T. Nguyen-Smith, A. Böddecker, L. Schücke, N. Bibinov, I. Korolov, Q.-Z. Zhang, T. Mussenbrock, P. Awakowicz and J. Schulze, *Plasma Sources Science and Technology,* 2022, **31**, 035008

2. L. Schücke, J-L. Gembus, N. Peters, F. Kogelheide, R.T Nguyen-Smith, A. R. Gibson, J. Schulze, M. Muhler and P. Awakowicz, *Plasma Sources Science and Technology*, 2020, **29,** 114003
